# Contrast-enhanced spectral mammography with a compact synchrotron source

**DOI:** 10.1371/journal.pone.0222816

**Published:** 2019-10-10

**Authors:** Lisa Heck, Martin Dierolf, Christoph Jud, Elena Eggl, Thorsten Sellerer, Korbinian Mechlem, Benedikt Günther, Klaus Achterhold, Bernhard Gleich, Stephan Metz, Daniela Pfeiffer, Kevin Kröninger, Julia Herzen

**Affiliations:** 1 Chair of Biomedical Physics, Department of Physics and Munich School of BioEngineering, Technical University of Munich, 85748 Garching, Germany; 2 Chair for Experimental Physics IV, TU Dortmund University, 44221 Dortmund, Germany; 3 Department of Diagnostic and Interventional Radiology, School of Medicine & Klinikum rechts der Isar, Technical University of Munich, 81675 München, Germany; University of Notre Dame, UNITED STATES

## Abstract

For early breast cancer detection, mammography is nowadays the commonly used standard imaging approach, offering a valuable clinical tool for visualization of suspicious findings like microcalcifications and tumors within the breast. However, due to the superposition of anatomical structures, the sensitivity of mammography screening is limited. Within the last couple of years, the implementation of contrast-enhanced spectral mammography (CESM) based on K-edge subtraction (KES) imaging helped to improve the identification and classification of uncertain findings. In this study, we introduce another approach for CESM based on a two-material decomposition, with which we expect fundamental improvements compared to the clinical procedure. We demonstrate the potential of our proposed method using the quasi-monochromatic radiation of a compact synchrotron source—the Munich Compact Light Source (MuCLS)—and a modified mammographic accreditation phantom. For direct comparison with the clinical CESM approach, we also performed a standard dual-energy KES at the MuCLS, which outperformed the clinical CESM images in terms of contrast-to-noise ratio (CNR) and spatial resolution. However, the dual-energy-based two-material decomposition approach achieved even higher CNR values. Our experimental results with quasi-monochromatic radiation show a significant improvement of the image quality at lower mean glandular dose (MGD) than the clinical CESM. At the same time, our study indicates the great potential for the material-decomposition instead of clinically used KES to improve the quantitative outcome of CESM.

## Introduction

At the present time, standard mammography is the state-of-the-art technology and an established screening method for breast imaging for women between the ages of 50 and 70 [1]. It is used for early breast cancer detection including the detection and classifications of microcalcifications, tumor nodules and cancerous lesions. Although the mortality rate has decreased since the introduction of mammography [2], further improvement is still desirable and necessary. This is underlined by recent reports about the sensitivity—a true positive rate which measures the proportion of actual positives that are correctly identified as positive—(between 69% and 94%) [3], the specificity—a true negative rate which measures the proportion of negatives that are correctly identified as negative—(between 78% and 95%) [3] and the positive predictive value which indicates how many people who have been diagnosed with a particular disease are actually ill (between 7% and 13%) [4].

The main issues in breast imaging, however, are the overlap of diagnostic relevant tissue structures in the case of women with dense breasts and the applied dose that is limited due to the radiation susceptibility of the breast [5]. Since the sensitivity decreases to 30-48% for women with dense breasts [6], cancerous lesions are sometimes not detected in standard mammography screening. Therefore, it is necessary to clarify suspicious, questionable or inconclusive mammography results with invasive procedures like biopsies or with time-consuming magnetic resonance imaging. In order to avoid these procedures, a method called contrast-enhanced spectral mammography (CESM), relying on K-edge subtraction (KES) imaging and the injection of iodinated contrast agent has been clinically used as quick check-up procedure of suspicious findings [7–10]. Two images—one with the mean energy below and one above the absorption K-edge of the iodine—are recorded to calculate an iodine image that shows the normal blood vessels as well as the newly formed blood vessels from the neoangiogenesis of the tumor [7–10]. The tumor nodules become visible, because there is a larger accumulation of blood vessels than usual. This method is currently only a check-up procedure due to the higher applied dose compared to standard mammography, the application of iodinated contrast agent and its possible adverse effects.

We propose to perform another approach for the iodine image calculation based on the dual-energy two-material decomposition method [11], for which we expect a better quantification of iodine contrast agent. With this approach, further spectral information can be exploited based on the characteristic property of the energy dependency of the linear mass attenuation coefficient. This method allows the discrimination of materials with similar mass attenuation coefficients like the commonly used iodine contrast agent and microcalcifications. Several studies on dual and spectral imaging approaches have already been developed and carried out at synchrotron facilities [12–15], but also with polychromatic sources spectral imaging and the distinguishability of these materials for coronary angiography has also been successfully demonstrated [16].

To fully exploit the potential of the proposed material-decomposition approach, the measurements of this study were performed at a brilliant compact synchrotron source called the Munich Compact Light Source (MuCLS). This source provides a quasi-monochromatic X-ray spectrum in an energy range which allows for eliminating X-ray photons mostly contributing to the applied dose and less to the image contrast. For a compact inverse Compton source (ICS), the X-ray energy is tunable, the X-ray beam is partially coherent and quasi-monochromatic. This additionally enables dose reduction while keeping the same image quality as with conventional X-ray tube sources used in clinical practice. The advantages of ICS compared to conventional X-ray tubes for mammographic applications have also been evaluated, showing a better performance based on the signal-to-noise ratio and the mean glandular dose (MGD) [17]. Thus, such a compact synchrotron source provides perfect conditions for our benchmarking study to demonstrate the highest achievable CESM performance. Various biomedical X-ray imaging applications like coronary angiography [18] or grating-based phase-contrast mammography [19] have already been successfully performed at the MuCLS, while a dose study of the latter shows that a significant dose reduction for biomedical applications is feasible [19].

Here, we propose the implementation of a dual-energy-based two-material decomposition approach for dose-compatible CESM. This study was conducted in combination with the MuCLS as a benchmarking study to show a proof-of-principle. With this innovation compared to the conventional, clinically conducted CESM procedure based on KES, we expect a significant improvement in image quality. Our results outperform the clinically available CESM method in terms of CNR, spatial resolution and MGD. Furthermore, we reduced the applied MGD by a factor of 60% at the same image quality (CNR and spatial resolution) as in clinical case. We are convinced that our material-decomposition approach has a great potential to replace the clinically used KES method for breast cancer detection.

## Materials and methods

### Phantom

**Fig 1:**
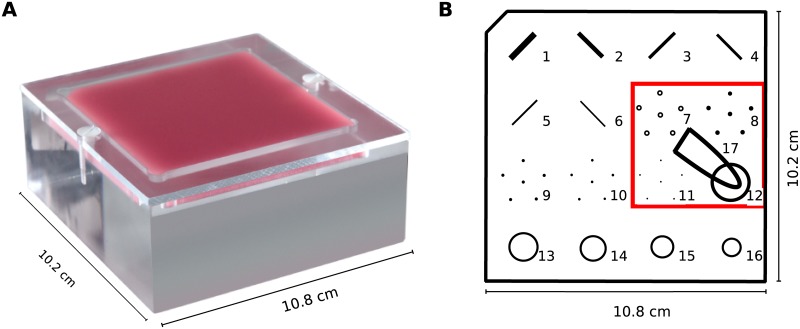
Mammographic accreditation phantom. It is shown as a photograph in (a) and as a scheme in (b) including different features as well as the iodine tube. The red box denotes the relevant area scanned in the experiments. Adapted from [20].

The measurements of this study were performed with a mammographic accreditation phantom (Gammex, Sun Nuclear Company, depicted in Fig 1) simulating a compressed breast composition of 50% glandular and 50% adipose tissue which contains microcalcifications, tumor masses and fiber structures of different sizes [20]. An Eppendorf tube with an iodine solution with a clinical concentration of 6 mg/ml was placed on the mammographic phantom to mimic the injected contrast agent. The size of tumorous lesions range from 1 mm up to more than 50 mm [21] depending on the stage of the tumor. The mean tumor size for breast cancer is about 2 cm [22, 23] and therefore the size of the Eppendorf tube is chosen to be approximately 1 cm x 3 cm. Due to the limited field of view, only the experimentally interesting area with microcalcifications, a tumor mass and the iodine tube is shown in the results.

### Clinical CESM examinations

**Fig 2:**
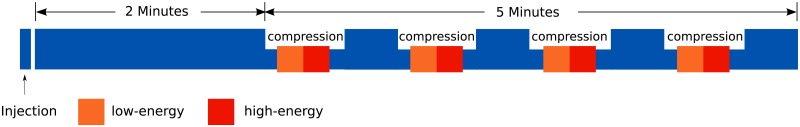
Schematic drawing of the examination procedure of clinical CESM [10]. Two minutes after the injection of the contrast agent, eight images in total are taken from different angles and both sides to get the recombined iodine image.

All clinical CESM examinations were performed with the SenoBright device by General Electric (GE) with a detector pixel size of 100 μm. The examination procedure for clinical applications is depicted in Fig 2. Two minutes after the injection of the contrast agent, the measurement begins and takes five minutes in total. However, our study was conducted with a static iodine capsule. For a better comparison between clinical and research setup, a dose study was performed. Therefore, images from 0.68mGy up to 4.07 mGy were acquired with manual exposure. For each measurement, two radiographs were taken: one with a mean energy below the K-edge with a tube voltage between 26 kVp and 30 kVp and one with a higher mean energy above the K-edge and a tube voltage of 45-49 kVp [10]. The exposure times and the filter combinations were adjusted individually to get images acquired with different dose levels. For the presented images, the associated X-ray tube settings are listed in Table 1. Finally, both images are automatically processed to a recombined iodine image which highlights the contrast agent and suppresses the surrounding background tissue. For medical diagnosis, the recombined iodine image and the low-energy image, which is comparable to a standard mammography image, are used.

**Table 1:** Image acquisition parameters for each measurement including the energy or tube voltage, the filter combination for the clinical measurement, the exposure times of the laboratory measurements, the tube current-exposure time product of the clinical measurement and the resulting applied MGD.

Modality	LE/ HE	Energy	Filter	Exposure time/tube current-exposure time product	Mean glandular dose [mGy]	Fig
Clinical	LE	27 kVp	Rh/Rh	56 mAs	0.76	4(a)
Clinical	HE	46 kVp	Rh/Cu	80 mAs	0.23	–
KES MuCLS	LE	25 keV	-	1.5 s	0.38	4(c)
KES MuCLS	HE	35 keV	-	2.0 s	0.56	–
MD MuCLS	LE	25 keV	-	2.5 s	0.64	4(e)
MD MuCLS	HE	35 keV	-	1.5 s	0.43	–

### Working principle of the MuCLS

The MuCLS is the first commercial installation of a compact synchrotron source based on inverse Compton scattering (developed by Lyncean Technologies Inc., USA) with a dedicated imaging beamline developed and installed by the Technical University of Munich [24]. This X-ray source aims to overcome the limitations of large-scale synchrotron facilities like high costs, limited availability and large sizes by combining their benefits such as the high brilliance with the relatively small size and the high availability of laboratory sources [25]. Electrons originating from a photocathode are subsequently accelerated to relativistic energies and finally stored in a miniature storage ring. They collide head-on with a counterpropagating infrared laser pulse which is circulating in an enhancement laser cavity. X-ray photons are produced in the interaction point where the electrons and the photon pulse collide with a frequency of about 65 MHz.

**Fig 3:**
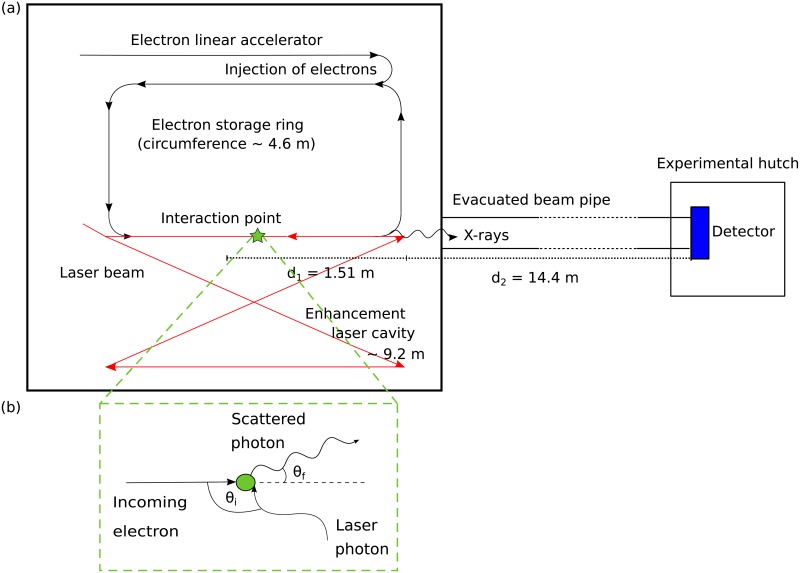
Schematic of the experimental set-up of the MuCLS (not to scale). (a) Schematic Layout of the MuCLS: The interaction of electrons, which are accelerated to relativistic energies in an electron linear accelerator, with a laser pulse stored in an enhancement laser cavity generates the X-rays. The detector in the experimental hutch is placed 16 m away from the interaction point. (b) Scheme of inverse Compton scattering.

The emitted energy of the X-rays *E*_*x*_ can approximately be calculated with *E*_*x*_ = 4*γ*^2^ *E*_*L*_, where *γ* = *E*_*e*_/*E*_0_ with *E*_0_ as rest energy and *E*_*e*_ total energy of the electron and *E*_*L*_ is the energy of the laser photons [24–26]. The working principle of inverse Compton scattering is illustrated in Fig 3. By adjusting the electron energy *E*_*e*_, the X-ray energy is tunable between 15 and 35 keV. The produced X-rays are emitted into an opening angle of 4 mrad, the X-ray beam is partially coherent and quasi-monochromatic. For an energy of 35 keV, the flux of the MuCLS—after a laser upgrade in spring 2017—is for example 3.0 ⋅ 10^10^ photons per second with a source size of less than 50 x 50 *μ*m^2^ [27]. In the experimental hutch, 16 m away from the interaction point, the beam has an elliptic shape of 62 mm x 74 mm (horizontal x vertical) [24].

### Image acquisition at the MuCLS

For the measurements at the MuCLS, a Dexela 1512 flatpanel detector (PerkinElmer Inc., USA) with a Gd_2_O_2_S scintillator and an effective pixel size of 71 × 71 *μ*m^2^ was used. For imaging of low contrast objects like in mammography, a flat-fat field correction should be performed with an equivalent or higher thickness of PMMA or water to approximate the X-ray material attenuation of the breast. Hence—in addition to the dark current correction—our images were also flat-field corrected with 6 cm PMMA in the beam path. Since the applied MGD was not known exactly before the measurement as the X-ray flux may fluctuate, a series of low- and high-energy images were initially acquired with different exposure times. The processing of the K-edge subtracted or material decomposed images followed after assessment of the MGD. The image acquisition parameters for the images presented in this work are listed in Table 1. The X-ray energy of the MuCLS was tuned to 25 keV (low energy) or 35 keV (high energy). The energy bandwidth is 3.60% and 4.29% and the full width at half maximum (FWHM) is 0.89 keV and 1.50 kev for the two energies, respectively [24].

### K-edge subtraction imaging at MuCLS

For dual-energy KES imaging with a conventional X-ray source, the energy is adjusted to an energy above and below the K-absorption edge directly after one another. Here, we perform a dual-energy KES where one radiograph is taken below the K-edge at 25 keV and one radiograph above the K-edge at 35 keV. Due to the energy dependency of the mass attenuation coefficient [28]

$$\begin{array}{c} \mu ∼\frac{1}{E^{3}}, \end{array}$$ (1)

it is necessary to make an empirical energy correction with (*E*_1_/*E*_2_)^3^ to realize a dual-energy KES. The images are then subtracted to obtain an image that only shows the contrast agent iodine.

### Two-material decomposition at the MuCLS

Since the measurements were performed with a quasi-monochromatic source, one can assume that the linear attenuation coefficient *μ*(*E*) is composed of the different linear attenuation coefficients of all materials which are included in the sample. For CESM, a discrimination of the injected contrast agent iodine and the microcalcifications is desired. To differentiate between these two materials, one decomposes the image in an iodine basis image and in a calcium basis image so that in one image *d*_*C*_ the microcalcifications and in the other image *d*_*I*_ the contrast agent is shown. For this decomposition, the following linear system of equations [11, 29] is solved

$$\begin{array}{c} \begin{array}{cc} \mu d(25\;\text{keV}) & =\mu_{I}\left(25\;\text{keV}\right)\cdot d_{I}+\mu_{C}\left(25\;\text{keV}\right)\cdot d_{C}\\ \mu d(35\;\text{keV}) & =\mu_{I}\left(35\;\text{keV}\right)\cdot d_{I}+\mu_{C}\left(35\;\text{keV}\right)\cdot d_{C}\;, \end{array} \end{array}$$ (2)

where *μd*(25 keV) and *μd*(35 keV) corresponds to the measured attenuation data of the respective energy, and *μ*_*I*_(*E*) and *μ*_*C*_(*E*) are literature values taken from [30]:

$$\begin{array}{c} \begin{array}{cc} \mu_{I}\left(25\;\text{keV}\right) & =68.796\;{\text{cm}}^{-1}\\ \mu_{I}\left(35\;\text{keV}\right) & =154.109\;{\text{cm}}^{-1}\\ \mu_{c}\left(25\;\text{keV}\right) & =10.650\;{\text{cm}}^{-1}\\ \mu_{c}\left(35\;\text{keV}\right) & =4.097\;{\text{cm}}^{-1} \end{array} \end{array}$$ (3)

After solving the linear system of equations, *d*_*I*_ and *d*_*C*_ represent the iodine and the calcium basis images, respectively.

### Dose calculation

For the clinical measurement with the SenoBright CESM developed by GE [10], the values for the MGD are automatically registered and listed for the recorded images in Table 1. For the quasi-monochromatic measurements at the MuCLS, an approximate dose calculation was performed to estimate the applied MGD. Therefore, the monoenergetic normalized glandular dose coefficients *DgN*(*E*) with *E* as X-ray energy tabulated by Boone et al. were used [31]. By taking the spectrum of the MuCLS [24] into account and summing over all energy bins *E*, the MGD can be calculated as follows

$$\begin{array}{c} MGD=\sum_{E}K\left(E\right)\left[mGy\right]\cdot 0.114[\frac{R}{mGy}]\cdot DgN\left(E\right)[\frac{mGy}{R}]. \end{array}$$ (4)

This formula is adapted from [31] but modified with the commonly used unit air kerma *K* as introduced by [19]. *K*(*E*) is the energy-dependent air kinetic energy released per unit mass (kerma), and *DgN*(*E*) are the monoenergetic normalized glandular dose coefficients depending on the thickness of the phantom and the assumed glandularity of 50%/50% (distribution of glandular and adipose tissue). The air kerma per energy bin for the MuCLS can be calculated with [32]

$$\begin{array}{c} K_{MuCLS}\left(E\right)=E\cdot \Phi \left(E\right)\cdot {\left(\frac{\mu_{en}}{\rho }\left(E\right)\right)}_{air} \end{array}$$ (5)

and depends on the mass energy attenuation coefficient of air (*μ*_*en*_/*ρ*)_*air*_(*E*) [33] and on the photon flux per energy bin Φ(*E*). It was calculated from a single photon-counting Pilatus 200K detector (Dectris Ltd., Switzerland) taking the X-ray spectrum of the MuCLS as well as the quantum efficiency of the silicon sensor of the detector [34] into account. For the correct calculation at the sample position, the distances between sample and detector are also considered. To allow for a precise calculation of the air kerma for each scan, the incident photon flux was monitored during measurements with a scintillation counter that had been cross-calibrated with the Pilatus detector. The calculation of the air kerma was validated by Eggl et al. with an X-ray ionization chamber (Model 34013, PTW Freiburg GmbH, Germany). In doing so, the measured and calculated values only differed by 10% [19].

### Image analysis

**Fig 4:**
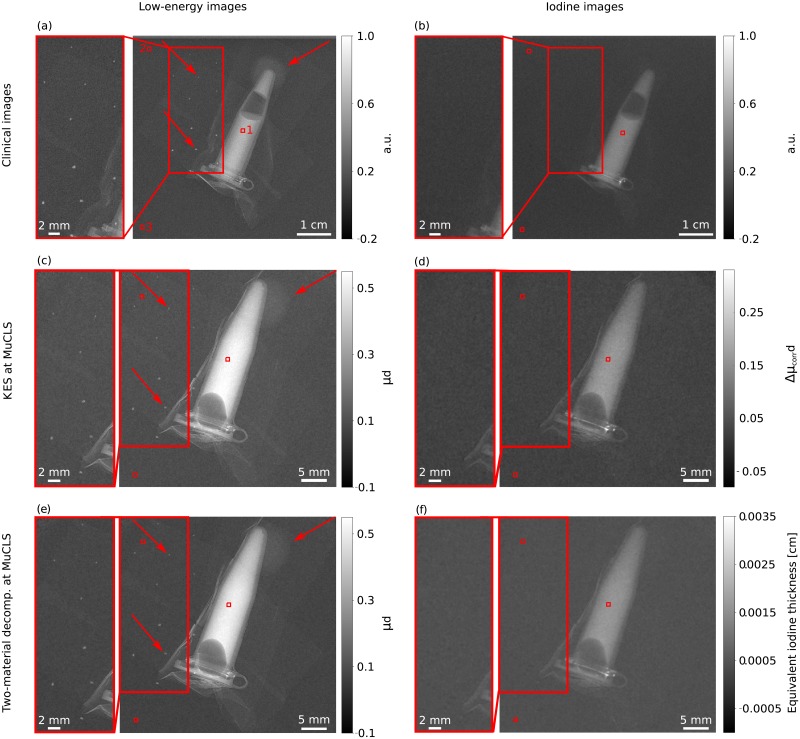
**Overview of the laboratory and clinical results of CESM** showing (a) the clinical low-energy image, (b) the clinical iodine image, (c) the low-energy image for KES at the MuCLS, (d) the iodine image processed with KES imaging, (e) the low-energy image of the material-decomposition at the MuCLS and (f) the iodine image processed with a two-material decomposition. Thereby, the zoom-in sections are intended to provide an improved representation of microcalcifications. The low-energy images in (a), (c) and (e) show the iodine tube, the microcalcifications and one tumor mass whereas in (b), (d) and (f) the successful elimination of the microcalcification and the tumor mass is shown and also underlined by the zoom-in sections. The microcalcifications and the tumor mass are indicated by the red arrows in Fig (a), (c) and (e). It should also be noted that as the images depict different quantities, a single common gray-level windowing suitable for all cannot be applied and the windowing is not the same in the presented images. The clinical images are given in arbitrary units and have only been normalized to one. All images were scaled for maximum detail visibility. The red boxes denote the different (ROIs) used for the CNR calculation of the iodine solution (cf. Eq 6).

In order to compare the results of the experimental work with the clinical CESM device, a dose-dependent calculation and analysis of the CNR and the spatial resolution was performed. The CNR is defined as the difference of two average signals $\bar{S_{1}}$ and $\bar{S_{2}}$ in two region of interests (ROIs) (indicated by the red boxes 1 and 2 in Fig 4, respectively) divided by the standard deviation *σ*_*BG*_ within a ROI of a background region (red box number 3 in Fig 4) as follows:

$$\begin{array}{c} CNR=\frac{\left(\bar{S_{1}}-\bar{S_{2}}\right)}{\sigma_{BG}}\;. \end{array}$$ (6)

The determination of the spatial resolution was performed with a power spectrum analysis [35]. After a Fourier transformation of the images, a Gaussian Filter with a kernel of 0.9 was applied to the squared norm of the Fourier transformation. The resolution of the images is then determined by the maximum spatial frequency where the spectral power of the signal corresponds the double the spectral power of the noise baseline, considering the effective pixel size of clinical and experimental images [35]. The calculated values for the image analysis for the presented results in Fig 4 are given in Table 2.

**Table 2:** Summary of the values for the spatial resolution and the CNR of the iodine solution of the presented results in Fig 4 also including the MGD for the respective low-energy and iodine images.

Modality	MGD [mGy]	CNR	Resolut. [lp/mm]	Fig
Clinical low	0.76	19.61±0.21	2.28±0.75	4(a)
Clinical iodine	0.99	22.80±1.42	2.23±0.61	4(b)
KES low	0.38	38.19±0.01	4.30±0.36	4(c)
KES iodine	0.94	25.34±0.21	5.14±0.21	4(d)
Mat. decomp. low	0.64	47.66±0.48	3.63±0.82	4(e)
Mat. decomp. iodine	1.07	28.65±0.11	4.65±0.35	4(f)

### Statistical analysis

**Fig 5:**
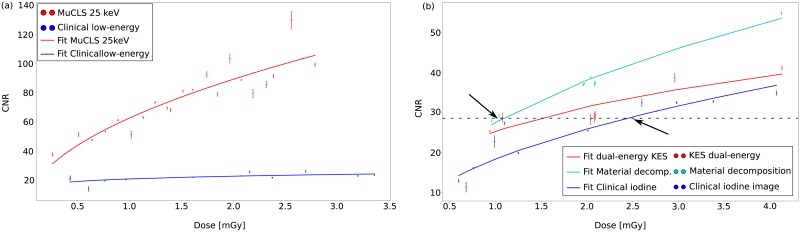
Dose-dependent overview of the CNR of the iodine solution for (a) the low-energy images and for (b) the iodine images acquired at the MuCLS and with the clinical SenoBright device. Thereby, the dots indicate the results of the measurements at different doses and the lines are the fitted root functions to the data to be able to see a tendency of the CNR with increasing dose. In both subfigures, it is clearly visible that the CNR of the laboratory results is better than of the clinical measurements with the SenoBright system. For the calculation of the iodine image, the two-material decomposition is the method with the highest CNR values. The possibility of reducing the MGD from clinically 2.5 mGy to 1 mGy is indicated by the black arrows and the intersection line in (b). The comparison of KES and material decomposition shows a reduction of 33% from 1.5 mGy down to 1 mGy which underlines the improvement of CESM with the material-decomposition approach.

To analyze whether there is a statistical correlation between the CNR and the MGD plotted in Fig 5, the Spearman’s correlation coefficient *r* for monotonous functions is calculated as well as a t-test is performed. For the t-test, which tests the significance of the statistical correlation, the t-value can be calculated as follows

$$\begin{array}{c} t-value=\frac{r\cdot \sqrt{n-2}}{\sqrt{1-r^{2}}}. \end{array}$$ (7)

In this equation, *r* is the correlation coefficient and *n* is the total number of observations. After checking the tabulated t-value, one can make a statement whether there is a statistical correlation between the data. The calculated values are listed in Table 3 in the results section.

**Table 3:** Statistical analysis of the correlation between the CNR and the MGD values from Fig 5. This includes the Spearman’s correlation coefficient r, the coefficient of determination r^2^ and the t-test. The calculated t-values exceed the tabulated t-values (for *α* = 0.05) so that a statistical correlation for the data is given.

	Clinical low	Clinical iodine	MuCLS low	MuCLS KES	MuCLS MD
Spearman’s correlation coefficient r	0.69	0.97	0.93	0.86	0.89
t-value calculated	3.04	11.18	11.58	4.07	5.87
t-value tabulated	2.282	2.262	2.080	2.447	2.262

To statistically analyze whether one metric is different to another (e.g. clinical vs. MuCLS low-energy images), we run a Kruskal-Wallis test on the different data sets. Therefore, we calculated the H-value

$$\begin{array}{c} H=[\frac{12}{n(n+1)}\cdot \sum_{j=1}^{c}\frac{T_{j}^{2}}{n_{j}}]-3\left(n+1\right) \end{array}$$ (8)

where *n* is the total number of observations, *n*_*j*_ the number of observation in group *j*, *T*_*j*_ the rank sums for the individual groups *j* and *c* the total number of observation groups. By comparing the experimentally calculated value with the respective *χ*^2^-value, we performed a statistical hypothesis test. The null hypothesis H_0_ was that the data sets do not differ from each other (no significant difference in CNR) and our alternative hypothesis H_1_ was that they differ (significant difference in CNR). The results are presented in Table 4.

**Table 4:** Results of the Kruskal-Wallis test to investigate whether there is a significant difference between the different methods. The null hypothesis can be rejected since the H-value exceeds the *χ*^2^-value in both cases.

Comparison	H-value	*χ*^2^-value
Low-energy images	23.00	3.84
Iodine images	8.03	5.99

## Results

In this dose-compatible study, the dual-energy material-decomposition approach as well as the clinically commonly used KES imaging method are conducted at the MuCLS and finally compared with the results of the CESM device (SenoBright, General Electric, USA) currently used in clinics. The measurements are conducted with a modified mammographic accreditation phantom (cf. Fig 1). In clinical routine, both the recorded low-energy image and the calculated iodine image are used for medical diagnosis. Consequently, both image types are presented and compared in this work. The results of the three different CESM measurements for a total MGD of about 1 mGy are presented in Fig 4. Quantitatively, they are evaluated using the CNR of the iodine solution in relation to a background region of the mammographic phantom, the spatial resolution and the MGD.

The results of the clinical measurements are depicted in Fig 4(a) and 4(b), the results of the dual-energy KES at the MuCLS are shown in Fig 4(c) and 4(d) and the results of the material-decomposition are presented in Fig 4(e) and 4(f). The low-energy images are shown in the left column and the iodine images in the right column. The zoom-in sections highlight regions of interests where microcalcifications are present. The low-energy images show the iodine tube, the microcalcifications and a tumor mass. The microcalcifications and one tumor mass are indicated by the red arrows. After the image calculation, all iodine images contain only the iodine solution, while the microcalcifications and tumor masses vanish. Hence, the separation of iodine and the microcalcifications works with all presented methods. It would be possible to calculate an image indicating a cancerous lesion by highlighting the newly formed blood vessels with an iodine-based contrast agent in all cases.

For a better evaluation of the achieved experimental results, a comparison between the clinical and the laboratory results, based on the CNR and the spatial resolution, is performed. In Fig 5(a), the dose-dependent CNRs of the low-energy images are plotted for the clinical and the MuCLS measurements. The low-energy images of the MuCLS have a CNR which is two to four times higher than the CNR of the low-energy images of the clinical measurement. Especially in the higher dose range, the values of both measurements differ significantly. Similar plots are presented in Fig 5(b) for the three different performed CESM methods, which are used for iodine image calculation. The results of the two-material decomposition approach outperform those of the KES imaging method at the MuCLS in terms of the CNR. The data were fitted as root functions. As quoted in Eq 6, the CNR depends on the variance. For our data based on f(I) = -ln(I/I_0_) the following applies to the variance

$$\begin{array}{c} \begin{array}{cc} \sigma_{d}^{2}=|\frac{\partial f(I)}{\partial I}{|}^{2}\cdot \sigma_{I}^{2} & =|-\frac{1}{I}{|}^{2}\cdot \sigma_{I}^{2}=\frac{1}{I}\\ \Rightarrow \sigma_{d} & =\frac{1}{\sqrt{I}}\;. \end{array} \end{array}$$ (9)

It follows that the CNR is proportional to the root of the intensity. For both image types (low-energy and iodine image), the spatial resolution is approximately between 3.5 and 5.2 lp/mm for the MuCLS measurements in contrast to the clinical measurement where the spatial resolution is between 2 to 3 lp/mm (cf. Table 2). Consequently, the spatial resolution of the MuCLS measurements is about twice as high as that of the clinical measurements due to the significantly smaller pixel size.

Based on the dose-dependent overview of the CNR, a significant MGD reduction from a clinical dose of 2.5 mGy to a laboratory dose of 1 mGy (indicated by the intersection line and arrows in Fig 5) using the dual-energy, two-material decomposition approach at the MuCLS was demonstrated. Additionally, a higher spatial resolution has been reached with the laboratory setups compared to the clinical CESM images (cf. Table 2).

A statistical analysis of the data plotted in Fig 5 is presented in Tables 3 and 4. The results of the Table 3 show that there is a significant correlation between the CNR and the MGD, since the experimentally calculated t-value exceeds the tabulated t-value for a significance level of *α* = 0.05 in all cases. This correlation is underlined by the correlation coefficient r. In addition, a Kruskal-Wallis test was performed to investigate whether there is a significant difference between the various plotted metrics. The first comparison includes the clinical versus the monochromatically acquired low-energy images. The second comparison deals with the possible differentiation of the three different iodine image calculation methods (clinical, KES at the MuCLS and material decomposition at the MuCLS). Since the H-value exceeds the *χ*^2^-value in both cases, the null hypothesis can be rejected. Thus, it has been shown that there is a significant difference between the plots in Fig 5(a) and 5(b).

## Discussion

Glandular breast tissue is known to be one of the tissues most sensitive to the applied dose. Hence, the goal of our benchmarking study was better quantification of the iodinated contrast agent to achieve the highest possible CESM performance at the lowest possible applied MGD with the material-decomposition approach instead of K-edge subtraction. We successfully demonstrate the high potential of the proposed two-material decomposition approach in this proof-of-principle study by using the quasi-monochromatic radiation of the brilliant and very compact MuCLS prior to transfer this approach to polychromatic X-ray sources. In addition, we also perform the commonly used KES to have a better comparison to clinical imaging. Both methods outperformed the clinical CESM procedure, but the two-material decomposition provides the most promising results in calculating an iodine image. On the one hand, at a comparable dose of approximately 1 mGy, the CNR is 25% higher than in clinical imaging and 15% higher than in the monochromatically acquired KES images. Thus, by increasing the image quality with another approach (material-decomposition) the dose can be significantly reduced while keeping the CNR constant. On the other hand, the CNR can be kept constant while reducing the dose by up to 60% comparing the material-decomposition approach with clinical imaging and by up to 33% by comparing the two monochromatically acquired images. This is illustrated by the intersection line in in Fig 5(b). The reduction of the applied dose is a very crucial factor when considering the radiation susceptibility of glandular breast tissue. Although the establishing of the novel X-ray source is not the focus of this work, we would also like to discuss the benefits of preclinical imaging with a novel X-ray source such as the MuCLS: We demonstrate a higher achieved spatial resolution at the same MGD compared to the clinical measurement (cf. Table 1). The significant improvement of the spatial resolution at the MuCLS, compared to the clinical measurements, is mainly caused by several factors like the smaller focal spot size and the smaller pixel size of the X-ray detector [36] at the MuCLS and its brilliance. Furthermore, we also showed that the low-energy images acquired at the MuCLS have a much higher CNR and spatial resolution than the clinically taken low-energy images. This is also very beneficial since the low-energy image is also used for medical diagnosis. With the improvement of the image quality providing the possibility to reduce the dose, it would also be possible to inject less contrast agent and keep the CNR constant and increase the spatial resolution. Especially for patients with adverse effects like hyperthyroidism [37] and allergic reactions [38] caused by the injection of iodinated contrast agent, a reduction of the injected contrast agent is desirable. Additionally, there is an increased risk for patients suffering from renal insufficiency, as this can lead to severe renal dysfunction and thus to dialysis treatment [37]. Until now, CESM is currently only a quick check-up procedure due to the injection of the contrast agent iodine and the higher applied MGD which is up to 70% higher than in standard mammography [39]. Due to that and based on the aforementioned improvements in our study, we are convinced that these results are significantly relevant to clinical practice.

One of the limitations of our study at the MuCLS was the time-consuming energy change of approximately 30 minutes during our experimental procedure. To become more practicable for clinical applications, a fast energy change would be necessary. Small energy changes with the machine can be performed reasonably fast (in some preliminary tests, changes by 1 keV have been performed in less than 5 s). However, as the power supplies of the magnets, which have to be adjusted for each energy change with the machine itself, take about 1-2 s to stabilize after a change of current, it will not be possible to reach the subsecond regime with this approach. For subsecond changes of the incident spectrum while leaving the source untouched, a filter-based approach was developed and successfully applied to K-edge subtraction imaging [40]. The energies currently adjusted to 25 keV and 35 keV could also be optimized. We are confident that an oscillation directly around the K-edge would further improve the contrast since the mass attenuation coefficient differs more around the K-edge. For synchrotron radiation, it has already been shown that an optimized energy separation of only 300 eV instead of 10 keV results in a 10,000 higher sensitivity for iodine compared with soft tissue [41]. Another drawback of the experimental setup is the limited beam size with which scanning larger biological samples would be necessary which consequently increases beam time. Since the field of view is currently only limited by the diameter of the exit aperture of the source, the beam size in the experimental hutch can be increased by employing a larger one. But since this is the first commercial installation of an ICS and this recently developed type of X-ray source is still strongly under research [42–45], we expect that further technical developments for inverse Compton sources will allow for an increased beam size, stability and flux as well as fast energy changes for dual-energy imaging enabling and improving prospective biomedical X-ray imaging. All in all and in contrast to the large-scale synchrotron facilities, a compact synchrotron source is more likely to be installed in a clinical environment as the spatial and the financial requirements are comparatively low.

Generally, our study mainly focuses on developing another approach rather than establishing this particular inverse Compton source as the next generation clinical X-ray source. Consequently, the material-decomposition approach should be demonstrated with polychromatic X-ray sources in further studies. In order to show an advantage for this procedure in clinical imaging compared to the commonly used CESM procedure, the measurements should be performed in one single image acquisition. Nowadays, spectral imaging with polychromatic X-ray sources can be realized in only one image acquisition by single photon-counting detectors where the impinging X-ray photons are directly converted into electrical charge. Biomedical X-ray imaging would benefit from the property of eliminating readout noise, but also from the energy discriminating property that allows the application of the spectral material-decomposition approach to polychromatic X-ray sources. For coronary angiography, a three-material decomposition has already been demonstrated [16]. But also by using other hybrid pixel detectors, spectral imaging has already been accomplished in several studies [46]. Hence, another possibility to achieve a higher CESM performance and a better quantification of the iodinated contrast agent is the combination of polychromatic X-ray sources with this detector technology. This is particularly interesting as it is expected that detector development will be faster than the technical development of compact synchrotron sources.

In conclusion, we were able to show that the two-material decomposition approach performed with an ICS for performing a proof-of-principle study is a promising alternative that significantly exceeds both the results of the clinical CESM device and those of the monochromatically performed KES in terms of spatial resolution and CNR. The quasi-monochromatic X-ray spectrum of the MuCLS additionally enables a dose reduction. Finally, we are confident that these advantages will become even more pronounced with future developments in the field of inverse Compton sources. Thus, we are convinced that the material-decomposition approach has a great potential to become an important tool in prospective medical diagnosis for breast cancer detection. Another interesting approach would be to investigate our method transferred to polychromatic X-ray sources using novel energy-resolving spectral detectors since with those detectors only one image acquisition would be necessary to perform a spectral material-decomposition.
